# Comprehensive Orthopedic Management of an Open-Book Pelvic Fracture: A Multidisciplinary Approach in Trauma Care

**DOI:** 10.7759/cureus.63669

**Published:** 2024-07-02

**Authors:** Mohamad H Saleh, Ahmed Elashmawy, Munna Hazime, Brandon Wallace, Mohamed A Saad

**Affiliations:** 1 Orthopedic Surgery, Wayne State University School of Medicine, Detroit, USA; 2 Surgery, Wayne State University School of Medicine, Detroit, USA; 3 Orthopedic Surgery, Corewell Health East, Dearborn, USA

**Keywords:** pubic rami fracture, open reduction internal fixation, high energy trauma, multi-disciplinary teams, crush injuries

## Abstract

Open-book pelvic fractures are an uncommon orthopedic emergency that requires prompt recognition and treatment. A 37-year-old male was involved in high-energy trauma, resulting in an open-book pelvic fracture with bilateral sacroiliac joint diastasis, bilateral superior and inferior pubic rami fractures, a comminuted sacral fracture, and a traumatic hernia. On presentation, he was hemodynamically unstable, with bruising in the right hemipelvis. Acute treatment included a cervical collar, transfusion protocol, central venous access, and pelvic binder. Trauma and orthopedic services were consulted to manage the patient with an interdisciplinary approach. The patient initially underwent external fixation with concomitant exploratory laparotomy. Definitive treatment concluded with colorectal anastomosis, diverting loop ileostomy creation, abdominal closure, open-reduction internal fixation (ORIF) of the pelvis, and removal and reapplication of external fixation.

## Introduction

In the realm of orthopedic emergencies, pelvic fractures are infrequent; even more so are open-book pelvic fractures, as demonstrated in our patient. Pelvic fractures constitute approximately 3% of all fractures but are associated with high mortality rates of 10.4% [1-4]. They commonly result from high-energy traumas such as motor vehicle collisions or falls from substantial heights. Early treatment, considered to be within the first four days post-injury, is crucial in mitigating the long-term complications of open-book pelvic fractures [4,5]. Among these complications, massive hemorrhage is the primary concern due to the rich vascularity of the pelvic cavity. Other challenges include pelvic girdle instability, which compromises weight-bearing capacity and mobility.

Open-book pelvic fractures, a specific subtype of pelvic ring injuries, are characterized by a disruption of the symphysis pubis and widening of the anterior pelvis, resembling an open book. This type of injury typically results from anteroposterior compression forces, often seen in high-energy trauma situations like motor vehicle accidents and falls from significant heights [6]. The severity of these fractures can vary, with the degree of pelvic ring disruption influencing both the stability of the pelvis and the extent of associated injuries.

Soft tissue injuries often accompany open-book pelvic fractures, adding to the complexity of these cases. These can include damage to the bladder and urethra, rectal and vaginal injuries, and nerve damage [7]. Specific nerves that can be injured include the lumbosacral plexus, particularly the sciatic nerve, femoral nerve, and obturator nerve. Blood vessels that may be affected include the internal iliac artery and its branches, which can lead to life-threatening hemorrhage [8].

The management of open-book pelvic fractures requires a multidisciplinary approach to address both the bony injury and the associated soft tissue damage. Hemodynamic stabilization, often through fluid resuscitation and blood transfusions, is a critical first step due to the high risk of hemorrhage. Surgical intervention may involve external fixation, internal fixation, or a combination of both, depending on the severity and stability of the fracture [9].

The rarity of pelvic fractures necessitates an individualized approach by orthopedic surgeons and trauma teams to minimize morbidity and mortality in these patients. In this case report, we present a detailed analysis of an open-book pelvic fracture with associated soft tissue injuries, highlighting clinical presentation, radiographic findings, management, and outcomes.

## Case presentation

A 37-year-old male presented to the emergency department (ED) after a 5,000-pound toolbox fell on his abdomen and lower extremities. Vitals upon arrival were blood pressure 92/52 mmHg, heart rate 76 beats per minute, temperature 97.4 °F (36.3 °C), respiratory rate 17 respirations per minute, and pulse oximetry at 96%. The primary assessment revealed a patent airway, normal breathing with a nonlabored rate and no retractions, normal and strong circulation, a Glasgow Coma Scale (GCS) score of 15 (eye 4, verbal 5, and motor 6), and no significant bleeding or gross deformities. The secondary assessment revealed a left inguinal bulge, generalized lower abdominal ecchymosis, and an abrasion in the right lower quadrant extending to the iliac crest. The abdomen was soft and distended. All other findings were normal. However, the patient's initial low blood pressure of 92/52 mmHg upon arrival to the ED likely stemmed from hemorrhagic shock due to significant internal bleeding associated with pelvic fractures and abdominal injuries. 

**Figure 1:**
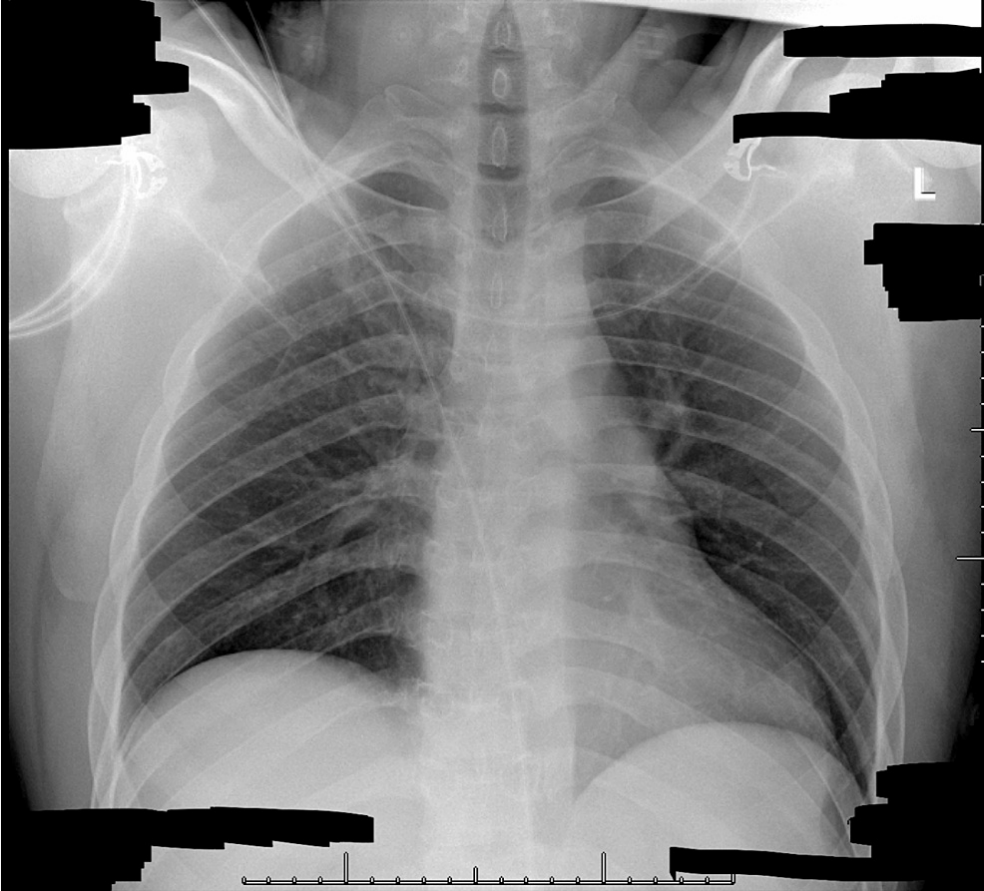
Portable chest radiograph. A single frontal view of the chest was obtained portably. The cardiomediastinal silhouette is within normal limits for technique. There is no focal consolidation, pneumothorax, pulmonary vascular congestion, or sizable pleural effusion. The visualized osseous structures are grossly intact.

**Figure 2:**
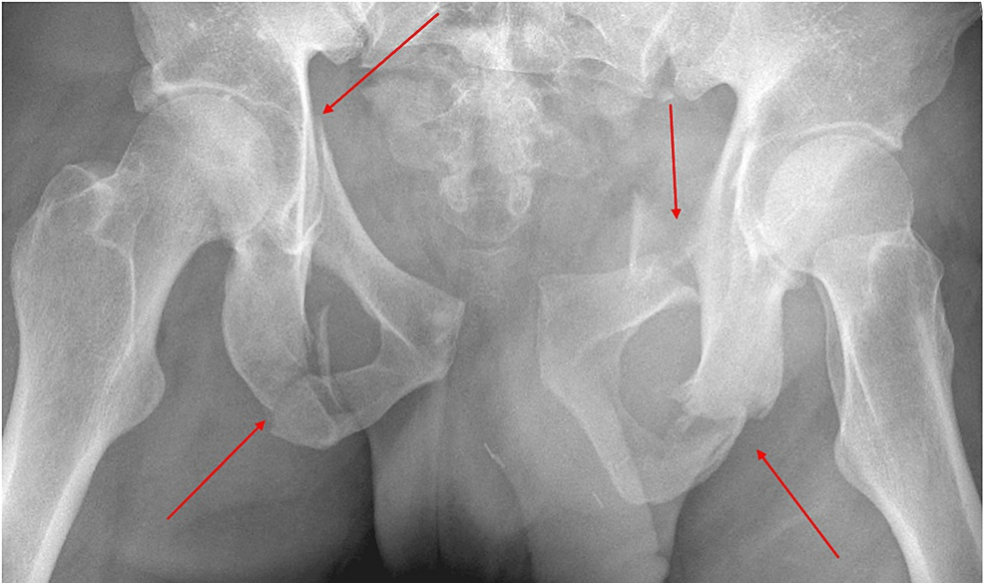
Pelvis radiograph. A single frontal radiograph of the pelvis was obtained. Markedly displaced fractures were noted through the bilateral superior and inferior pubic rami with gross distraction of the pubic rami sacroiliac joint. Distraction was also suspected, with fractures extending through the S3 vertebral body. Points of fracture are indicated with red arrows.

**Figure 3:**
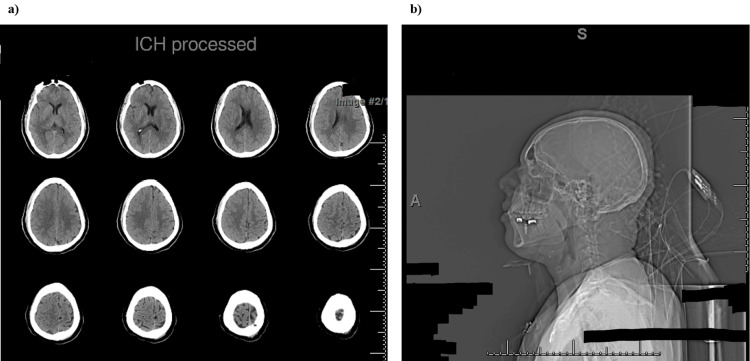
CT of the head and cervical spine. (a) Non-enhanced axial CT imaging through the head shows no evidence of intracranial hemorrhage (ICH) or cerebral edema. Gray-white matter differentiation is preserved. The ventricles are normal in size. The basal cisterns are patent. The calvarium is intact. (b) Non-enhanced CT imaging through the cervical spine shows maintained cervical vertebral body heights. There is no evidence of acute fracture. There is also no prevertebral edema or paraspinal hematoma. Spinal alignment is normal.

Due to the mechanism of injury, a chest X-ray was performed to assess for pneumothorax, hemothorax, and rib fractures (Figure 1). A pelvic X-ray was ordered to assess for pelvic fractures and significant internal bleeding (Figure 2). The Focused Assessment with Sonography for Trauma (FAST) exam at bedside was negative for fluids in Morison's pouch, the pericardial window, the perisplenic view, and the Pouch of Douglas. The negative findings on the FAST exam, which indicated no intraperitoneal or pericardial fluids, guided further imaging studies in order to identify the source of bleeding and direct subsequent management. Laboratory studies were performed in the ED (Table 1) and demonstrated metabolic acidosis, renal impairment, liver injury, and muscle damage. A computed tomography (CT) scan of the head without intravenous (IV) contrast, alongside a CT of the cervical spine, was performed immediately after the X-rays to evaluate for traumatic brain injury, as well as fractures, dislocations, and compression of the spinal cord or nerve roots caused by fractures (Figure 3).

**Table 1:** Relevant laboratory results upon admission to the ED. The elevated glucose suggests hyperglycemia, while the increased WBC count indicates an inflammatory response. RBC count, hemoglobin, and hematocrit are all decreased, suggesting severe blood loss. Electrolyte imbalances are seen through elevated chloride and decreased CO_2_, indicating metabolic acidosis. Renal function is impaired as seen by the elevated creatinine and reduced eGFR. The low albumin indicates hypoalbuminemia from protein loss. The critically high creatine kinase and lactic acid levels indicate muscle damage and severe lactic acidosis, respectively. Urinalysis showing hematuria with hyaline casts suggests significant renal compromise. *An abnormally low result. **An abnormally high result. U/L, units per liter; hpf, high-power field; lpf, low-power field

Test	Result
Glucose	176 mg/dL**
ABO/RH type	O+
WBC	13.3 billions/L**
RBC	4.04 trillion/L*
Hemoglobin	11.4 g/dL*
Hematocrit	36.4%*
Sodium	140 mmol/L
Potassium	4.5 mmol/L
Chloride	112 mmol/L**
CO_2_	17 mmol/L*
Anion gap	11
BUN	16 mg/dL
Creatinine	1.72 mg/dL**
eGFR	52 mL/minute/1.73 m^2^*
PT	13.3 s
aPTT	25.0 s
INR	1.2
Albumin	3.2 mg/dL*
CK	3,074 U/L**
Lactic acid	9.2 mmol/L**
Urinalysis color	Yellow
Urinalysis clarity	Cloudy
Urinalysis glucose	Negative
Urinalysis bilirubin	Negative
Urinalysis ketones	Negative
Urinalysis specific gravity	1.027
Urinalysis blood	2+**
Urinalysis pH	6.0
Urinalysis protein	30 mg/dL
Urinalysis urobilinogen	1.0 mg/dL
Urinalysis nitrites	Negative
Urinalysis leukocyte esterase	Negative
Urinalysis RBC	>20/hpf**
Urinalysis WBC	0-5/hpf
Urinalysis casts, hyaline	3-5/lpf**
Urinalysis bacteria	Negative

**Figure 4:**
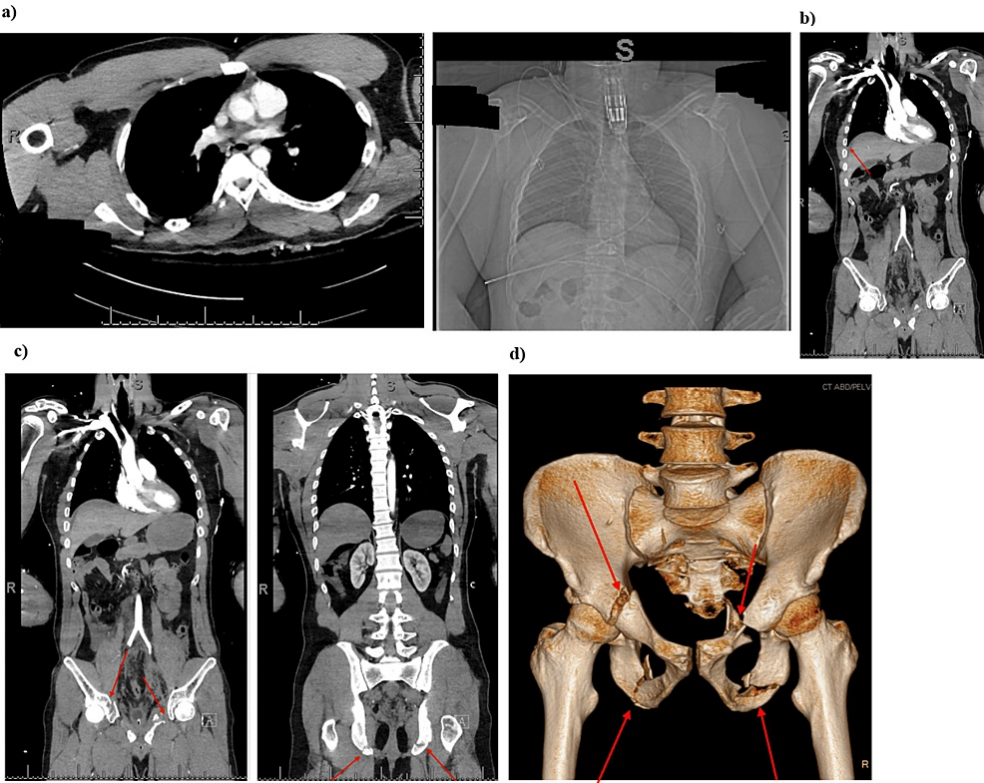
CT scans of the chest, abdomen, and pelvis. Axial and coronal CT images of the thorax, abdomen, and pelvis were obtained after the uneventful administration of IV contrast. One hundred milliliters of Isovue 370 was injected. (a) No cardiomegaly or pericardial effusion present. There is no mediastinal lymphadenopathy. There is no evidence of aortic dissection or thoracic aortic aneurysm. There are no hilar or adnexal masses. There is no focal airspace consolidation, pleural effusion, or pneumothorax. There is no acute displaced rib fracture. Sternum is intact. Thoracic vertebrae appear intact. (b) Free fluid noted within the pelvis and presacral space and the paracolic gutters. Hyperdense fluid noted within the extraperitoneal pelvis and intraperitoneal locations. The abdominal aorta is nonaneurysmal. Free air is noted within the abdomen. Underlying perforation to hollow viscus given consideration (indicated by red arrow). The presence of free fluid in the pelvis and intraperitoneal space on CT imaging raised concern for possible associated bladder or urethral injuries, necessitating ongoing monitoring and potential intervention. (c) There are extensive pelvic fractures. Fractures are noted involving the left superior and inferior pubic rami. Fractures are noted involving the right acetabulum and right inferior pubic ramus. There is also a fracture involving the right superior pubic ramus. No femoral head dislocation. No osseous fractures noted involving the sacrum posteriorly. Blood products found within the intraperitoneal and extraperitoneal pelvic space. Points of fracture are indicated with red arrows on CT. (d) 3D rendering isolating the skeletal abnormalities. Sites of fractures are indicated by red arrows.

**Figure 5:**
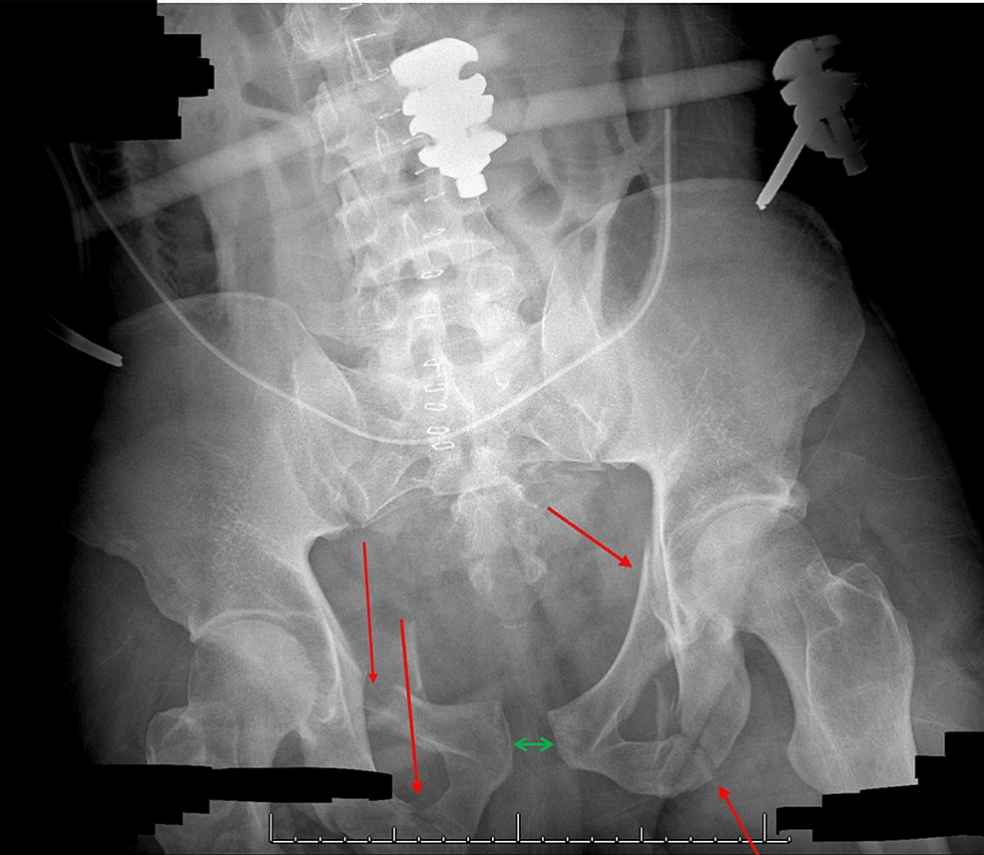
Pelvic X-ray of the external fixture before open-reduction internal fixation. Right superior and inferior pubic rami fractures are seen with minor displacement. There is a left superior pubic ramus fracture without significant displacement and a comminuted, mildly displaced fracture of the left inferior pubic ramus. Additionally, there is pubic symphysis diastasis (indicated by green line) and very mild widening of the sacroiliac joints. An external fixation device is noted. Points of fracture are highlighted by red arrows.

Due to concern for spinal cord injury, the absence of prevertebral edema or hematoma on cervical spine CT and normal spinal alignment suggested no acute spinal cord injury requiring immediate intervention. Finally, CT scans of the chest, abdomen, and pelvis were ordered to evaluate internal organs, bones, and blood vessels and identify any injuries not visible on X-ray or FAST (Figure 4). Once the patient was stabilized in the ED with blood, plasma transfusions, and IV fluids, he was transported to the operating room where he underwent an exploratory laparotomy with small bowel resection, segmental resection of the descending colon, placement of a temporary abdominal closure, and a pelvic external fixture (Figure 5). Although the specific quantities of blood products administered were not detailed, the need for exploratory laparotomy, segmental bowel resection, and subsequent operative management implied significant blood loss requiring multiple units of packed red blood cells and plasma.

**Figure 6:**
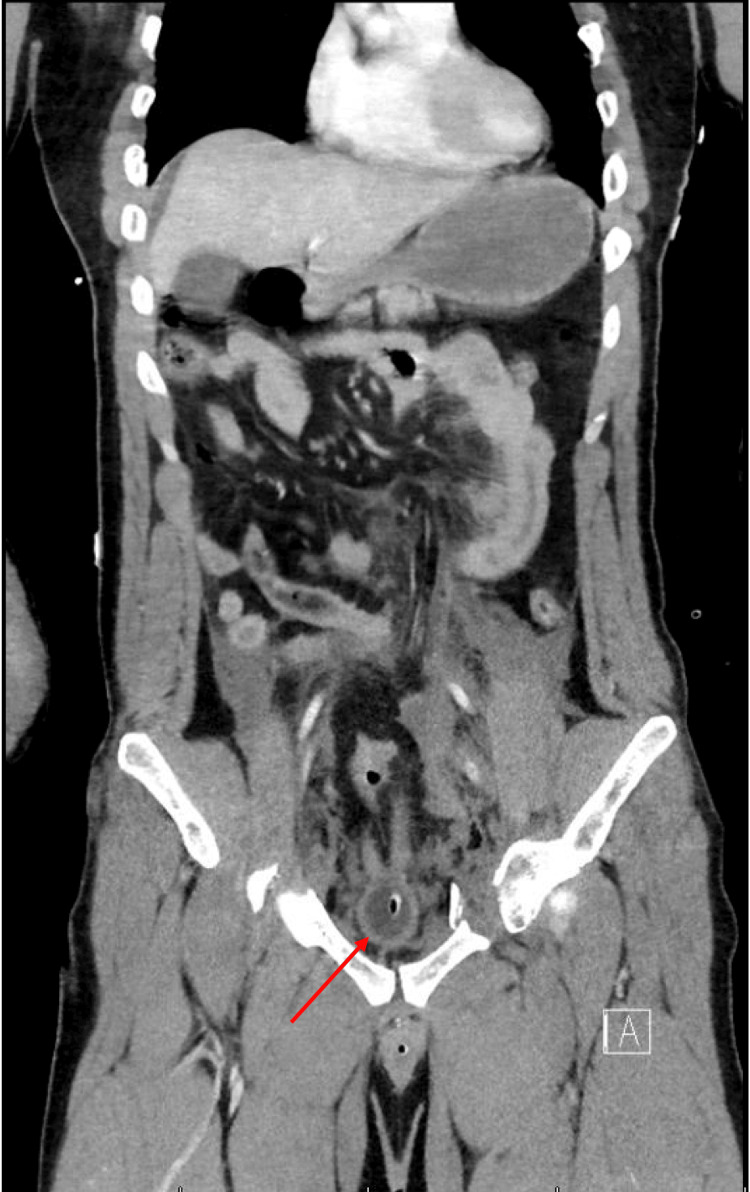
Abdominal CT scan highlighting the bladder. Coronal abdominal CT scan revealing an extraordinarily compressed bladder, which could have been the result of a potential bladder rupture, especially in the context of a pelvic crush injury or open-book pelvic fracture. The compression of the bladder suggests that it has been subjected to substantial external pressure, likely from displaced pelvic bones or direct traumatic forces. This degree of compression increases the risk of bladder wall rupture. Furthermore, the presence of free fluid in the retroperitoneal space on the CT scan supports the diagnosis of a ruptured bladder, as urine may leak into this space following a tear in the bladder wall. Note: The bladder is indicated by a red arrow.

The decision for damage control surgery/exploratory laparotomy following initial stabilization in the ED was indicated by ongoing hemorrhage and the need for bowel resection and pelvic fixation. This approach was aimed at controlling the bleeding and preventing further deterioration. Following the exploratory laparotomy, which involved extensive abdominal exploration and bowel resection, a pelvic binder was then used to stabilize pelvic fractures. This was done to control ongoing hemorrhage from pelvic vascular structures and to facilitate subsequent orthopedic interventions, such as open-reduction internal fixation (ORIF), by reducing pelvic volume and maintaining alignment. The possibility of bladder rupture was also considered due to findings on preoperative imaging (Figure 6).

**Figure 7:**
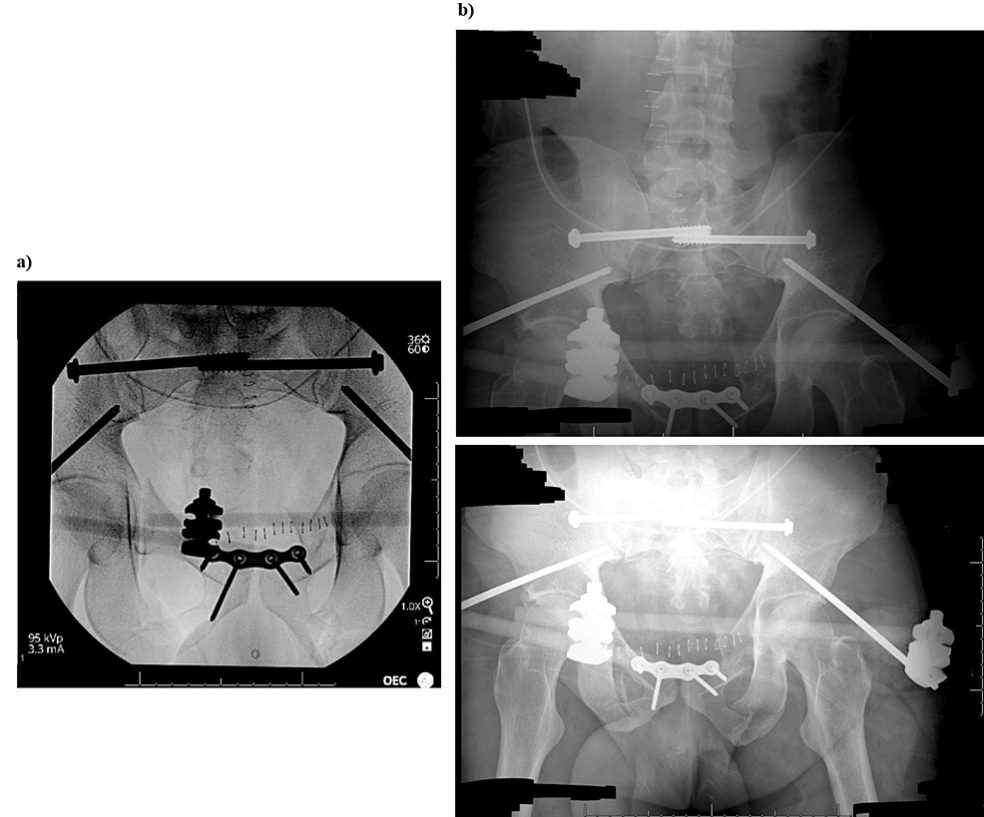
Pelvic X-rays of the external fixture after open-reduction internal fixation (ORIF). (a) Pelvic X-rays obtained using procedural fluoroscopic guidance during hardware fixation of the previously described multiple pelvic fractures. (b) Status post-ORIF of the pelvis with satisfactory anatomic alignment of the fractured fragments and no unexpected radiopaque foreign bodies seen. Overlying hardware appears intact.

On the following day, the trauma surgeons performed a second-look abdominal exploration with wash-out, colorectal anastomosis, diverting loop ileostomy creation, and abdominal wound closure. Once he was stable from a trauma perspective, the orthopedic surgeons were able to conduct an ORIF of the pelvis with reapplication of the external-fixture device and placement of bilateral sacroiliac screws (Figure 7).

## Discussion

Open-book pelvic fractures are uncommon and pose a significant challenge to orthopedic surgeons and trauma teams due to their infrequent occurrence. This limitation prevents the development of a standardized protocol for open-book pelvic fractures across all ages. For instance, a 14-month-old girl with an open-book pelvic fracture due to a motor vehicle accident had total resolution with an external fixator [10]. Conversely, this treatment was the initial step in rectifying our 37-year-old male patient.

The preferred imaging modality for open-book pelvic fractures is CT with contrast. This allows visualization of arterial bleeding with a sensitivity of 60% to 90% and 87% to 98% [11]. However, after fluid resuscitation, CT with contrast was completed following a portable frontal radiograph of the pelvis and a FAST exam, the latter of which was negative, underscoring the importance of additional imaging in a hypotensive patient. Further CT scans without contrast were also performed to gauge the integrity of the brain and the spinal cord. This challenges current literature stating that delayed surgical management of pelvic fixation leads to worse long-term outcomes [4]. The full assessment was necessary given the parallel traumatic hernia. The orthopedic intervention began with a pelvic external fixture but was delayed until the trauma team addressed the hernia.

The management of this patient can be summed up in four steps: hemorrhagic control, initial pelvic stabilization, soft tissue resolution, and definitive pelvic fracture treatment with open reduction and internal fixation. The order of steps 2-4 varies in individual cases, but in the observation of another case with a pelvic fracture and soft tissue injury, the latter was resolved before the pelvic fracture. This case involved a 29-year-old male patient admitted following a motor vehicle accident who presented with generalized pelvic pain and right testicular displacement [12]. The testicular displacement was a distracting injury that delayed the treatment of the pelvic fracture. An investigation into how much time has elapsed between soft tissue and pelvic fracture resolution, whether it was prioritized or missed, is necessary to determine whether this delay is significant in the recovery of individuals with pelvic fractures.

To maximize the potential outcome for treatment, orthopedic and trauma teams worked in tandem to provide timely care. Despite the definitive treatment of the pelvic fracture occurring six days following admission, total care for both the traumatic hernia and fracture concluded one week after admission. This timeline was in part due to a fluid line of communication necessary to maintain comprehensive care.

In this particular instance, due to the high mortality rates associated with open-book pelvic fractures, this patient will have to be monitored closely after his operation as this injury is associated with complications such as avascular necrosis of the femoral head, venous thromboembolism (VTE), and surgical site infection [13-15].

While the management strategy employed here aligns with protocols at Level 1 trauma centers globally, this case underscores the importance of a multidisciplinary approach, timely imaging, and the order of surgical interventions tailored to individual patient needs. The use of a pelvic binder, although a common practice, played a crucial role in the initial stabilization post-exploratory laparotomy, highlighting its utility in complex trauma care scenarios. The coordination among various specialties contributed to the successful stabilization and subsequent recovery of the patient, reflecting best practices in trauma care and the necessity for continuous evaluation and adaptation of treatment protocols based on individual patient presentations and outcomes.

## Conclusions

These findings collectively highlight the severe impact of high-energy trauma on multiple organ systems, emphasizing the need for an integrated and multidisciplinary approach to trauma care. The interplay between orthopedic surgeons, trauma teams, and other medical specialists is paramount in managing complex injuries like open-book pelvic fractures with associated soft tissue damage. Open communication and coordination are crucial to minimize postoperative complications and ensure optimal patient outcomes.

In this case, we demonstrated the importance of early recognition of injuries, prompt and effective resuscitation, and timely intervention. The management strategy included initial stabilization with a pelvic binder and external fixation, which was critical in controlling hemorrhage and maintaining pelvic stability. Subsequent surgical interventions addressed both the pelvic fractures and associated soft tissue injuries, highlighting the necessity of prioritizing life-threatening conditions while planning for comprehensive care.

While the management techniques employed in this case align with established protocols at Level 1 trauma centers worldwide, the detailed account of the collaborative approach and the sequencing of interventions provides valuable insights into best practices. This case underscores the need for continuous evaluation and adaptation of treatment protocols based on individual patient presentations and outcomes.

Our objective was to illustrate the role of a multidisciplinary team in managing complex trauma cases, reaffirming the principles of trauma care while highlighting the nuances of individual case management. By sharing this case, we aim to contribute to the ongoing dialogue on optimizing trauma care and improving outcomes for patients with severe, multisystem injuries.
